# Morel paper is a passionate plea for the concept of “translation” with translational research (TR) as a flag

**DOI:** 10.1590/0074-02760210277chgsb

**Published:** 2022-04-15

**Authors:** Jean Jannin

**Affiliations:** 1 Président de la Société Francophone de Médecine Tropicale et Santé Internationale

It clearly demonstrates, if necessary, that bridging scientific advances, public health and political commitment is the necessary condition for success of control programs and for, in the best case, for reaching elimination. It would seem logical to think that this may sound something so very obvious based on some simple questions:

- Who can affirm that research is not a major determinant of the medical breakthrough and a powerful tool for reducing the burden of poverty?

- Who can be enough intellectually impaired to think that each research achievement as modest as it is, do not need to be immediately translated into action, integrated into public health policies and made accessible to all.

- Who could imagine that research doesn’t take place in a coherent and effective approach together with medical science, social science and public health?

- Who can think that TR is not taking a prominent part in the necessary reconciliation of science and politics?

The answer to these questions should undoubtly been: Nobody! And yet it is!

Carlos Morel is providing the response in the last paragraph: Politicians and bureaucrats of all kind…

The concept of innovative developing countries (IDCs) developed here is not only primordial, but represents a goal to be reached by many countries and more represents an important step on the way to become part of Developed Countries.

This concept is also including the idea of effective networking and shared capacity building. With this aim in mind, IDCs need to play a crucial role in supporting LDCs in their efforts of research capacity building.

But let us never forget that fighting neglected tropical diseases (NTDs) is the responsibility of all countries without exception, simply because ensuring public health and reducing the burden of these diseases at the minimum is an ethical duty and a fundamental human right. Diseases are a universal concern, and not just the problem of those who are infected and consequently not the problem of the only affected countries. This dangerous idea is sometimes heard from (LDCs) explaining that the problem of diseases affecting a country must be solved by itself! Of course, this discussion is only occurring when allocation of funds is on the agenda.

In the same vein, this distinction is often polluting the debate between giving priority to communicable or non-communicable diseases and surprisingly, Least developed countries (LDCs), affected by NTDs, are very often promoting the second one… Each one need to play his part actively for finding solutions.

All countries are full of brilliant people and all this brilliant youth must be at work in the best possible conditions. There are not enough capacities in the world to afford to waste brainpower!

It’s one of the principles of the concept of IDCs.

The need for eradicating clannishness in the scientific community based on the proudness or supposed superiority of various research teams is one of the corollaries of the TR. Each excessive importance given to one specific part (mainly through excessive funding) is weakening the other parts of the chain and subsequently creates neglect and inefficiency. In the area of neglect, there is no room for waste!

Successful programs are the result of a subtle equilibrium between all components. If we agree to go far beyond the product development, the idea continues to work. That’s the beauty of the concept of TR as well as the necessity of breaking boundaries between various sciences enabling all multifactorial components to be considered. The “One Health” concept is moving and is an illustration of the added value of the “spillover” concept.

As mentioned, the establishment of product development partnerships (PDPs) as well as public private partnerships (PPPs) were and are a powerful way to be free from the shackles of bureaucracy or privileged interests.

But, as in any human business, self-services motives are often persisting and governments for their part are happy to transfer their responsibilities for a fistful of dollars to non-governmental entities.

It is reminded in the article that TR is the most effective way to produce new tools in a minimum of time. We need to keep in mind that the “magic tool” doesn’t exist! The mirage of a tool able to definitely stop diseases remain a dream among politicians who prefer to invest on highly sophisticated illusions than on effective evidence-based know-how!

A good tool is the one for which a universal access is ensured and provide significant progress!

Carlos Morel is reminding the emblematic story of the Chagas disease since 1909 to 1979 in Brazil and how these efforts were successful, but also how these achievements are fragile and fully dependent of unjustified financial cuts… But political will has persisted during the last three decades and consolidation of control of Chagas disease has been made effective through the establishment of four intergovernmental initiatives in Latin America and one in non-endemic countries (NEC) to ensure the global cover of Chagas disease control and the commitment of governments. The disease was included in the official list of NTDs in 2005 by the World Health Assembly (WHA). A WHA resolution was adopted (WHA 63-20) in 2010.

Drugs have been made available mainly for pediatric formulation… Since 2012, the WHO NTD roadmap, endorsed by the WHA, is providing milestones and goals in the fight against the disease. A World Chagas Day was established in 2019.

These elements added to the progress discussed above are contributing to secure the capacities to fight the disease, even if very counter-productive decisions are taken at country level, like in Brazil, one of the most advanced country for research and control of Chagas disease.

As Sysiphus pushing his rock to the top of the mountain, generations of passionate people tried to eliminate diseases and particularly NTDs. As Sysiphus they never succeeded in stabilising the rock on the peak, and this one, as their disappointed hopes tumbles down…

All of us, never abandoning, are taking the rock and push it again and again, convinced that perseverance and conviction pay off.

The task is not lost in advance and intelligence and boldness will help the community to convince politicians to be consistent in their efforts and never abandon so close to the victory!
